# Neuromuscular Retraining versus BTX-A Injection in Subjects with Chronic Facial Nerve Palsy, A Clinical Trial 

**DOI:** 10.22038/ijorl.2021.41305.2347

**Published:** 2021-05

**Authors:** Abbas Ali Pourmomeny, Elham Pourali, Ahamd Chitsaz

**Affiliations:** 1 *Department of Physical Therapy, School of Rehabilitation Sciences, Isfahan University of Medical Sciences, Isfahan, Iran.*; 2 *Department of Neurology, School of Medicine Sciences, Isfahan University of Medical Sciences, Isfahan, Iran.*

**Keywords:** Botulinum toxin A, Facial nerve palsy, Neuromuscular retraining, Synkinesis

## Abstract

**Introduction:**

Chronic facial nerve palsy has long been known to negatively affect the quality of life in patients. The present study aimed to investigate the efficacy of Botulinum Toxin A (BTX-A) and neuromuscular retraining therapy (NMRT) in the symmetry of chronic facial palsy.

**Materials and Methods:**

Two groups, namely experimental and control, were considered each consisting of 13 patients. The study population included a total of 15 female subjects. The BTX-A was injected into the synkinetic muscle in the experimental group; nevertheless, the patients in the second group participated in special neuromuscular retraining. The rate of reducing synkinesis and symmetrical improvement was evaluated using the Facial Grading System (FGS) after 4 months of treatment. The independent t-test was performed to compare the two groups.

**Results:**

According to the Sunnybrook FGS, the mean changes in Sunnybrook scores in the experimental and neuromuscular retraining groups were 3% and 24%, respectively. The comparison of the mean scores of the two groups was statistically significant after the interventions (P=0.033). A variable ratio of statistically significant improvement was observed in synkinesis in both groups following the treatment (P=0.041). In addition, by comparing the synkinesis scores between the two groups, the reduction of synkinesis was observed to be greater in the neuromuscular retraining group (P=0.041) after the treatment.

**Conclusions:**

The findings of the current study indicated that special neuromuscular retraining leads to significant improvement in the FGS score, compared to botulinum toxin therapy alone. Moreover, it was observed that facial symmetry can be treated with special neuromuscular retraining and the patient can overcome synkinesis.

## Introduction

Synkinesis, muscle tension, muscle contracture, and atrophy are the most common consequences of chronic facial nerve palsy (FNP) (1). Muscle tension refers to the condition in which the muscles of the face remain semi-contracted for an extended period. Muscle contracture is a permanent shortening of a muscle or soft tissue. As an involuntary and abnormal facial movement associated with voluntary movements of other facial muscles, synkinesis is thought to be the result of misdirected sprouting, re-innervating both the original and other facial muscles (2). It has been estimated that synkinesis is diagnosed in around 15-20% of individuals depending on the case series (3,4). The appearance and progression of synkinesis may each require 3 to 4 months and up to 2 or more years, respectively (5). Accordingly, the manifestations of FNP can range from muscular contracture to tension and weakness. Furthermore, synkinesis disrupts muscular coordination and causes an asymmetrical connection between the two facial opposite sides. All four sequelae cause asymmetry in the face at rest and dynamic movements and especially the standard expression of the face is affected which significantly impacts patients’ quality of life. The application of precise and specific feedbacks during a process known as facial neuromuscular retraining can help to retain and relearn facial movements. 

In addition, the aforementioned process can be beneficial in preventing unwanted muscular movements and acquiring normal facial expression, patterns, and functionality. In a previous study, BTX-A was studied as a complementary therapy for facial symmetry with biofeedback training (6). 

The present study aimed to compare the effectiveness of injecting BTX-A and (NMRT) using electromyographic biofeedback (EMG‐BFB) when BTX-A was infused into synkinesis site or patients underwent 4-month NMRT with long-term facial palsy.

## Materials and Methods

This clinical trial was registered in the Iranian Registry of Clinical Trials (IRCT201612136083 N10). The Ethics Committee of Isfahan University of Medical Sciences, Isfahan, Iran, also approved the study protocol under the code of IR.MUI.REC.1395.3.128. 

The study population was recruited by referring to physiotherapy clinics. The subjects with peripheral facial paralysis were initially examined by three specialists (including a plastic surgeon, an ear, nose, and throat specialist, and a neurologist). The inclusion criterion was an incomplete recovery after one year, the development of unilateral peripheral facial paralysis complications, and the diagnosis of synkinesis on the affected side. The exclusion criteria were having BTX-A hypersensitivity, being pregnant or in the lactating period, and suffering from either peripheral motor neuropathies or neuromuscular junction disorders, such as myasthenia gravis. 

The eligible subjects were requested to give informed consent by signing a pre-prepared form. Then, the patients were randomly allocated to either the experimental (BTX-A) or control (NMRT) group. For randomization, each pair of participants was consecutively (one member in each pair was assigned to one group leading the other member to the opposite group) and randomly assigned to either group upon admission. Each patient was monitored within 8 to 12 weeks to confirm the constancy of synkinesis, and then the treatment was started. Finally, within May 1 in 2015 to May 3 in 2019, 26 patients (including 15 female and 11 male subjects) had completed treatment in the study. Table 1 shows the demographic and clinical features of the participants.

2-1. Injection

The asymmetry, synkinesis, and tension were carefully examined in the first stage. The synkinetic points were identified on the patient’s face. Then, normal saline (2.5 mL) was used to dilute BTX-A vials containing 300 units of the compound. 

**Fig1:**
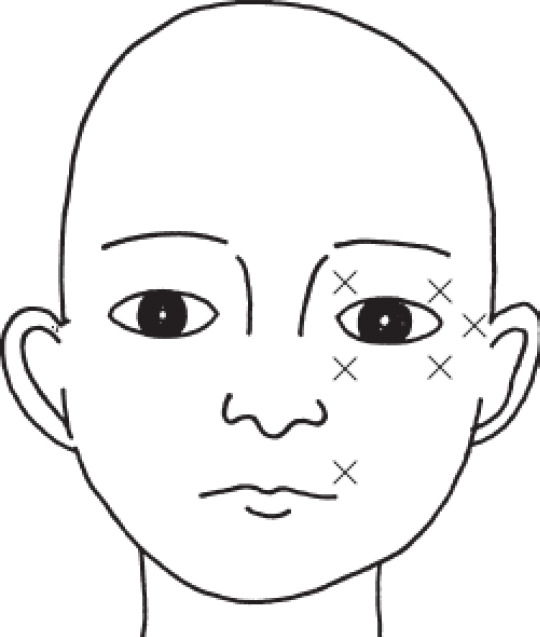
Locations for facial injections of Dysport

The diluted toxin was then infused into the synkinetic muscular tissues at three to seven sites (Figure 1). The synkinesis muscles around the eye were injected with 10 to 15 units at each point, and the synkinesis muscles around the mouth were injected with 15 and 20 units. The injections were administered using an insulin syringe (7).

**Table1:** Demographic and clinical features

Variable	BTX-A^**^	NMRT^***^	P-value
**Age ** (mean±standard deviation)	43.67±10.5 years	37.67±14.5 years	
**Gender** Male Female	4 9	7 6	
**Side** Left Right	11 2	7 6	
**Cause** Bell’s palsy Trauma Tumor (Schwannoma)	12 0 1	11 2 0	
Average number of treatments	1	49±3	
Average total cumulative dose; units (per case)	125	-	
Average duration of disease (month)	43.93±67	34.42±34	0.66
FGS*	45.14±18.2	39.83±16.2	0.97

* Facial Grading System; ** Botulinum toxin type A; *** Neuromuscular retraining therapy

2-2. NMRT

All the standard expressions were analyzed by a specialized physiotherapist, and the weak, contracted or tense, and synkinetic muscles were determined in each patient. In this study, a normal saline solution, as a placebo injection, was injected into the members at several points of the affected side. The NMRT included massaging the soft tissue, stretching the contracted muscles of the affected side, relaxing the tense muscles, and giving EMG‐BFB for muscle strengthening and synkinesis control. After providing the patients with some preliminary data on the anatomy and various muscles of the face, along with their functions, during the 4-month treatment period, three 45-min sessions were performed each week. The participants were also taught how to control synkinesis and movement. Therefore, mirror biofeedback as home training was prescribed for each patient.

2-3. Outcome measures 

The subjects’ facial characteristics were assessed at the baseline (before the treatment) and after the 4-month treatment period utilizing videotapes in the rest position of the face and five standard expressions. Three skilled experts who were unaware of the patients’ group assignments independently assessed the videotapes before and after the treatment. The degree of facial symmetry (in resting position, dynamic movement, and synkinesis) was evaluated by a score in the Sunnybrook Facial Grading System (FGS) ^(6)^. The SPSS software (version 16; SPSS Inc., Chicago, IL, USA) was utilized for data analysis using the two-tailed test at 0.05 as the level of significance. The patients were examined for complications, such as ptosis, at (after) a two-week post-injection.

## Results

The baseline features of the two groups were homogeneous and showed no statistically significant difference (P=0.66; Table.1). Three patients suffered ptosis and drooling for a short time (about 10 days). The FGS composite scores and subdomains were calculated for resting symmetry, symmetry of voluntary movement, and synkinesis symmetry in both groups. In the BTX-A group, the total FGS composite scores were 45.14±18.2 and 48.36±17.5 before and after the treatment, showing an increasing but insignificant pattern (P=0.51), respectively. In comparison, the FGS composite score of the NMRT group increased from a mean of 39.83±16.2 to 64.17±10.4 (P<0.01) in the post-treatment group. The difference in the composite scores between the two groups was statistically significant after the treatment (P=0.033). The FGS subdomain synkinesis in both groups significantly improved. The scores of subdomains of voluntary movement and resting symmetry in the BTX-A group were 66.5±15.2 to 67.14±15.3 and 11.43±3.6 to 10±3.9, respectively (Table.2), which were not statistically significant (P=0.08). The scores of subdomains of voluntary movement and resting symmetry in the NMRT group were 63±13.8 to 75±11.4 and 12.08±2.5 to 6.67±3.8, respectively, which were statistically significant.

**Table 2:** The results of comparing two treatment

**Group**	FGS*: Pre- treatment Mean ± standard deviation				FGS: Post- treatment Mean ± standard deviation			
	Rest	Voluntary movement	synkinesis	FGS	Rest	Voluntary movement	synkinesis	FGS
BTX-A	11.43±3.6	66.5±15.2	10±2.6	45.14±18.2	10±3.9	67.14±15.3	7.01±2.3	48.36±17.5
NMRT^**^	12.08±2.5	63±13.8	11.08±2.5	39.83±16.2	6.67±3.8	75±11.4	4.17±2	64.17±10.4
*p-value*	0.23	0.94	0.87	0.97	0.83	0.035	0.041	0.033

* Facial Grading System; ** Botulinum toxin type A; *** Neuromuscular retraining therapy

## Discussion

This has been the first study that has directly compared two treatments in patients with the sequelae of FNP. According to FGS analysis, facial nerve synkinesis was successfully managed and resolved in both groups, further confirming literature findings (6,8) . Despite the decreased synkinesis scores in both groups, a lower mean score in the BTX-A group may be due to its therapeutic halftime in this group. In addition, only the FGS composite score and voluntary movement in the MNRT group increased significantly. This result is a logical consequence of active exercise, stretching, and relaxation procedures in NMRT. 

The purpose of BTX-A injection is to reduce or eliminate synkinesis and indirectly modify the asymmetry in facial movement. Then, 3-10 days after using BTX-A injection, synkinesis was eliminated through the inhibition of acetylcholine release at the synapse level. Following this 4-5 month period, one of the following conditions may occur: 

1) synkinesis totally vanishes; 

2) synkinesis may partially relapse; 

3) synkinesis may completely relapse and appear again. 

It has been suggested that the infusion of BTX-A into the normal facial side may be effective in retaining symmetry (9-11). However, the asymmetry of facial palsy will not be corrected and the asymmetry may increase (12). 

Given the delicacy and small size of facial muscles, they are referred to as the superficial musculoaponeurotic system (13). The voluntary and facial expression movements are performed in a pattern with minimal contraction. It is important to accurately identify the synkinesis and weak or tough muscles before treatment. It has to be noted that the analysis of facial movements is essential for the success of NMRT. 

The goal of NMRT using biofeedback in FNP is enhancing the quality of movement and motor control and upgrading muscular functionality to resolve the asymmetry. Another important point is to convince patients to accept and try to treat their defect. This asymmetry either is a result of muscle weakness, increased muscular tension, or consequence of synkinesis. 

The EMG‐BFB facilitates neuromuscular re-education and allows providing patients with the opportunity to perceive surrounding auditory and visual signals which are naturally hidden to the patient. Through monitoring the activity of motor units during this process, the patient is also able to control the function of a muscle or a group of muscles. Using these feedback approaches, the patient is enabled to create a partial movement following taking the control of a number of motor units. Furthermore, patients can be taught how to suppress muscular motor activities (e.g., synkinesis and tension) due to a sequel of facial palsy. This phenomenon is due to the lack of proprioception producing internal sensory receptors in facial muscles (6,14). 

In addition and unlike skeletal muscles, there are no facial shields for the closure of muscles in the face (6). 

Contrary to some studies (15,16), the patients in the present study were in a socially active age range (mean age: 42 years). The participants had a high motivation for recovery, particularly in the NMRT group, with an average age of 37 years. The results of the current study showed that the injection of BTX-A decreased synkinesis; nevertheless, it could not affect the weakness and contraction of the muscles. Some researchers suggest BTX-A injections more than one session for the reduction of synkinesis. However, further investigations and comparisons are necessary in this regard.

## Conclusion

Although BTX-A injection may reduce synkinesis, it cannot affect the voluntary movement in FNP with just one session of treatment. Nonetheless, NMRT is the rational treatment for muscle weakness and synkinesis caused as the sequelae of FNP.
